# Procedure-Related Mortality in Aspiration Thrombectomy for Pulmonary Embolism: A MAUDE Database Analysis of the Inari FlowTriever and Penumbra Indigo Systems

**DOI:** 10.1177/15266028241307848

**Published:** 2024-12-23

**Authors:** Blair E. Warren, Kong Teng Tan, Arash Jaberi, Laura Donahoe, Marc de Perrot, Micheal C. McInnis, John T. Granton, Sebastian Mafeld

**Affiliations:** 1 Department of Medical Imaging, Temerty Faculty of Medicine, University of Toronto, Toronto, ON, Canada; 2 Division of Vascular and Interventional Radiology, Joint Department of Medical Imaging, University Health Network, University of Toronto, Toronto, ON, Canada; 3 Division of Thoracic Surgery, Toronto General Hospital, University Health Network, University of Toronto, Toronto, ON, Canada; 4 Division of Cardiothoracic and Vascular Imaging, Joint Department of Medical Imaging, University Health Network, University of Toronto, Toronto, ON, Canada; 5 Division of Respirology, Department of Medicine, University Health Network, University of Toronto, Toronto, ON, Canada; 6 Peter Munk Cardiac Centre, Toronto General Hospital, University Health Network, University of Toronto, Toronto, ON, Canada

**Keywords:** embolism, endovascular treatment/therapy, thrombectomy, thrombosis, thrombus

## Abstract

**Background:**

Pulmonary embolism (PE) is an important cause of death and disability. Advances in catheter-directed therapies have led to the use of devices, such as the Inari FlowTriever and Penumbra Indigo system for aspiration thrombectomy (AT) for both massive and sub-massive PE. However, limited data exist on causes of procedural mortality.

**Methods:**

Analysis of the Food and Drug Administration’s (FDA) Manufacture and User Facility Device Experience (MAUDE) database was performed. Data for the Inari FlowTriever and Penumbra Indigo aspiration thrombectomy systems were evaluated for mortality events and classified by cause of death from January 1, 2015, to December 31, 2023.

**Results:**

The review identified 26 mortality events related to the Inari FlowTriever and 28 related to the Penumbra Indigo device. Pulmonary vascular perforation (n=26) and right heart injury/tamponade (n=9) were the most common source of mortality. Clot migration (n=4) and acute right heart failure (n=5) were less frequently observed.

**Conclusions:**

This study reveals more mortality events than have been captured in the literature to date. Vascular perforation and cardiac injury are the most common and also potentially preventable sources of mortality. Strategies to mitigate complications related to aspiration thrombectomy are described.

**Clinical Impact:**

Analysis of mortality in aspiration thrombectomy (AT) for acute pulmonary embolism is necessary to better understand the safety profile of this procedure. This analysis of the MAUDE database reports the largest single cohort of 54 deaths. Potentially preventable procedure-related mortality in AT has been documented to be the result of vascular perforation and cardiac perforation with tamponade. Preparation for emergent pericardiocentesis should be considered in mechanical thrombectomy. Clot migration may result from thrombus maceration or migration of clot in transit, thus, careful pre-procedure examination for clot in transit with echocardiography is suggested.

## Introduction

Pulmonary embolism (PE) remains an important cause of death, with high-risk PE mortality reaching rates of up to 20.6% in contemporary data.^1^ There is a trend toward use of catheter-directed therapies for both high-risk/massive and intermediate-risk/sub-massive PE and in particular the use of aspiration thrombectomy (AT), as it avoids the inherent risks associated with systemic pharmacologic thrombolysis. The 2 dominant devices at present are the Inari FlowTriever system (Inari Medical, Irvine, California) and the Penumbra Indigo system (Penumbra, Alameda, California).^2–6^ Remarkably, despite the large diameter of these devices, there are very few reported mortality events or complications in the literature associated with the use of these devices.^4,5,7,8^ For example, in the Inari FlowTriever All-Comer Registry for Patient Safety and Hemodynamics (FLASH), zero vascular injuries or device-related mortality events within 48 hours were reported and only 1 cardiac injury among 788 patients was identified.^5^ Similarly, Penumbra’s Evaluating the Safety and Efficacy of the Indigo aspiration system in Acute Pulmonary Embolism (EXTRACT-PE) study found only 1 device-related death within 48 hours, 1 pulmonary vascular injury, and zero cardiac injuries among 119 patients.^4^ This is in contrast to a recent real-world data that report higher mortality ranging from 3.5% to 6.7% mortality.^9,10^ If AT is to become standard of care, procedural-related and device-related mortality must be well understood for patient selection, consent, and technical success. Real-world data may demonstrate other sources of device-related or procedure-related complications that are of value for operators planning to use AT.^11^

The United States Food and Drug Administration’s (FDA) Manufacture and User Facility Device Experience (MAUDE) database is a valuable resource that captures medical device event data, with nearly 3 million events recorded in 2022.^12^ Highlighting the utility of the MAUDE database, a 2022 clinical consensus statement on percutaneous treatment options for acute PE included an analysis of MAUDE data for several different devices for the treatment of PE, however, only 4 deaths related to AT were captured at that time.^3^ Another study examining the Penumbra Indigo aspiration system found only 2 deaths.^11^ As more time has elapsed since the FDA approval of these devices for PE, an interim analysis of the MAUDE database may reveal new data to aid operators in understanding the risks and benefits of these devices in the treatment of PE. It was hypothesized that AT-related mortality events in the FDA’s MAUDE database would provide insight into the types of AT complications that result in mortality. With this knowledge, strategies to avoid or recover from complications could be developed.

## Methods and Materials

Data were queried from the MAUDE database with search dates from January 1, 2015, to December 31, 2023, which precedes the approval dates for both devices to ensure inclusion of all data. Inari device data were obtained through the web interface of MAUDE, by searching “Inari” in the manufacturer (accessed February 2, 2024). Penumbra’s larger portfolio of devices required a search using the MAUDE API via Python (Python Software Foundation) by searching for manufacturer “Penumbra” and event text containing “pulmonary,” which is not possible using the MAUDE web interface (accessed February 7, 2024). All event texts from the retrieved data were manually reviewed and all events resulting in mortality were included in the analysis. Only mortality events were examined, as there is a paucity of data on procedural-related or device-related mortality in the literature. Specific device malfunctions, such as wires breaking or failure of hemostatic valves, were not a point of interest in this study.^11^ Irrelevant (eg, not pulmonary thrombectomy or non-thrombectomy device-related), duplicate, and non-mortality events were removed from the data. After initial review of the data, a classification system for the types of complications leading to mortality was developed and all mortality events were then classified by authors B.E.W. and S.M. Due to the nature of MAUDE data, a denominator is not known for device-related events, and therefore, descriptive statistics alone are reported. For the purposes of this study, manufacturer devices are analyzed as a whole, that is, all Penumbra Indigo catheter sizes are included in Penumbra (7-, 8-, 12-, and 16-French sizes) and the same for Inari (16-, 20-, and 24-French sizes). Due to the nature of MAUDE data that may sometimes be incomplete, some results were deemed indeterminate. Patient clinical details and demographics are not known in MAUDE and therefore not included. Ethics approval was waived as the study uses public data.

## Results

**Figure 1.**
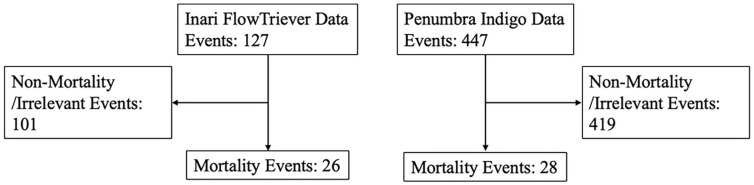
Study cohort flow diagram denoting the MAUDE data retrieval and cleaning.

Inari data retrieved 127 events with 26 deaths and Penumbra 447 events with 28 deaths (Figure 1). Types of complications leading to mortality were: pulmonary vascular perforation, right heart injury and/or tamponade, clot migration, acute right heart failure (aRHF), and indeterminate (Tables 1 and 2). One case of paradoxical embolism was also documented. Pulmonary vascular perforation was the most common AT complication resulting in mortality in the MAUDE data, with 26 events documented. The second most common complication was right heart injury and/or tamponade with a total of 9 events seen. Acute right heart failure was seen in 5 cases and clot migration in 4 cases. One case of a paradoxical embolism through a patent foramen ovale was observed in a procedure using the Inari device. Within the Penumbra cohort, 4 of the deaths also incidentally noted breakage of the Separator device, which is used to disrupt thrombus, with no clear relationship to mortality. One aRHF death in the Inari cohort was in part related to blood loss (reported 660 mL) in a comorbid patient with a do not resuscitate order. The indeterminant deaths in both cohorts had no clear identifying cause in the event description. No reported deaths were from intracranial hemorrhage.

**Table 1.** Count of Complications Associated With Mortality for the Inari FlowTriever and Penumbra Indigo System Captured in the MAUDE Database.

Complication	Inari FlowTriever	Penumbra Indigo system	Total (n)
Pulmonary artery perforation	10	16	26
Right heart injury/tamponade	5	4	9
Clot migration	3	1	4
Acute right heart failure	4	1	5
Paradoxical embolism	1	0	1
Indeterminate	3	6	9

**Table 2.** Classification of Mortality Associated Complications Captured in the MAUDE Database With More Than 1 Event.

Complication	Description	Strategy to avoid complication
Pulmonary artery perforation	Vascular perforation either by the wire, sheath, or mechanical thrombectomy device. This includes instances of intra-procedural hemoptysis, which were likely related to vascular perforation including when explicitly stated or evident based on the narrative included (eg, angiogram showed contrast extravasation into the lung parenchyma)	Use of a stiff wire for support with a short floppy tip (eg, 1 cm floppy tip). Need for innovation in guidewire technology
Right heart injury/tamponade	Injury to the right heart including acute pericardial effusion resulting in tamponade. Note that it is possible this captures main pulmonary artery vascular injuries near the pulmonic valve, where it is covered by pericardium	Consider less traumatic strategies for crossing the right heart, including pigtail, angled pigtail, and balloon-tip catheter. Avoid pushing with resistance. A balloon can be inflated and passed over the wire to ensure no resistance. Ensure safe wire position and support. Avoid manipulating the aspiration thrombectomy without a wire or dilator. Caution applying repeated aspiration if against a vessel wall
Clot migration	Change in embolic distribution or rapid progression of pulmonary embolism, either by migration from within the pulmonary vasculature or from clot in transit (eg, right atrial thrombus)	Pre-procedural echocardiogram can be performed to rule out right atrial thrombus. If there is migration of thrombus prompt aspiration may be required based on clinical deterioration
Acute right heart failure	Rapid hemodynamic collapse associated with pulmonary embolism as captured in the report	Proceed with aspiration thrombectomy in a prompt fashion. Consider adjunctive supportive measures based on local availability and access (eg, right ventricular support devices or extracorporeal membrane oxygenation)

Not included is a single case of paradoxical embolism via a patent foramen ovale.

## Discussion

Aspiration thrombectomy is growing as a method of treating not only high-risk/massive PE in the rescue setting, but also intermediate/sub-massive PE as a means of shock prevention.^2,3^ The current analysis captures a greater number of mortality events than any of the existing literature on AT using the Inari or Penumbra devices in the FLASH, FLAME (FlowTriever for Acute Massive PE), or EXTRACT-PE studies, as 30-day all-cause mortality in those studies only captured 10 deaths.^4,5,7^ More specifically, only 1 device-related death was reported in those studies versus the 54 herein, and therefore, the causes of mortality related to AT procedures may be under-reported. This study identifies the types of events resulting in mortality in AT, which are critical to know to aid with patient selection, consent, and procedural safety. Vascular perforation and right heart injury were the most common causes of mortality. Vascular perforation or injury is a well-known complication of any pulmonary arterial procedure.^13^ Indeed, the EXTRACT-PE study of the Penumbra device found 2 instances of pulmonary vascular injury.^4^ The pulmonary vasculature is subject to extreme movement in the setting of tachypnea, therefore, caution should be taken with positioning the stiff wire required for delivery of these large bore devices into a deep position and constant verification of wire position under fluoroscopy is a necessity (Table 2). If we assume that pulmonary artery perforation is linked to the guidewire tip, this could happen with both devices. When considering differences between the devices, one to consider is the step-off between the wire and the catheter in the Penumbra system as it historically does not come with a dilator like the Inari system. In this study, we do not know if operators using the Penumbra system used catheters to reduce the step-off. The manufacturer has recently introduced a catheter with up to 98% lumen occupancy to reduce the step-off, which theoretically could reduce trauma. The impact of French size is also a difference to consider, as the Inari is up to 24-French compared with the Penumbra 16-French, and it is unknown whether the lumen occupancy or stiffness has an impact on right heart and pulmonic valve function. This may be a consideration in determining specific etiologies of aRHF.

Strategies to reduce vascular injury, such as pre-curving the 1 cm short taper Amplatz Super Stiff wire (Boston Scientific, Marlborough, MA, USA) have been suggested, but no “perfect” guidewire providing the necessary stiffness/support for large bore device advancement through pulmonary arterial anatomy with an atraumatic distal tip currently exists. Resulting pulmonary artery hemorrhage or injury can be treated by balloon occlusion, embolization, or intubation and bronchial blockers if expertise is available.^13^ However, intubation is frequently avoided in acute PE as it may precipitate aRHF due to reduced preload and increased afterload.^14^ Right heart injury and resulting tamponade can similarly be prevented by careful wire manipulation and verification of a non-subvalvular or sub-chordae path of the wire by passing a balloon freely over the wire. A pericardiocentesis kit should be readily available at the time of AT to treat potential tamponade.

Clot migration is a rare but known possibility when manipulating catheters in the setting of PE.^2,11^ This may be the result of thrombus migrating from within the pulmonary arteries more distal, thus resulting in immediate increase in the pulmonary pressures. Alternatively, disruption of thrombus in the inferior vena cava (IVC) or right heart, known as “clot in transit,” can result in acute migration of thrombus into the right ventricular outflow tract or pulmonary arteries.^2,15^ Careful pre-procedural planning with an echocardiogram can facilitate detection of clot in transit and can aid in planning AT prior to instrumenting the right heart. The instances of aRHF in this study are likely partially explained by clot migration or acute decompensation with the device in situ, which can result in decompensation in the setting of hemodynamically significant PE in these fragile patients, however, insufficient detail is provided to establish the clear clinical cascade.^2,16^ Ultimately, AT, catheter-directed thrombolysis, or systemic thrombolysis are all an attempt to prevent aRHF, thus, this is a known intra-procedural complication. Finally, only 1 death was thought to be in part related to intra-procedural blood loss (660 mL), as AT does rely on aspiration of blood volume, which is not always returned depending on the device.

There is still much to be learned about who, how, and when to best treat acute PE patients with AT. A recent study examining ultrasound-assisted catheter-directed thrombolysis and AT found contemporary in-hospital mortality rates for PE treated by these endovascular methods to be 2.9% and 3.5%, respectively.^9^ The Pulmonary Embolism Thrombolysis (PEITHO) trial saw a reduction in death or hemodynamic collapse from 5.6% (28 of 499) in the placebo group to 2.6% (13 of 506) in the tenecteplase group.^17^ However, the tenecteplase group also saw significantly more hemorrhagic complications than the placebo: 10 cases of hemorrhagic stroke and 32 cases of extracranial bleeding in the tenecteplase group versus 1 and 6 in the placebo group. Therefore, if endovascular methods, such as AT result in a similar reduction in mortality similar to thrombolysis but without the hemorrhagic risk, it is conceivable that it may become standard of care. Studying causes of mortality in AT is of utmost importance and the results of this study outline the main reported causes of intra-procedural or peri-procedural death, with vascular perforation and right heart injuries accounting for the highest number of reported deaths. Operator experience and careful wire manipulation may mitigate these risks.^6,13^

## Limitations

Limitations of the study are related to the nature of the MAUDE database in that the overall incidence of injuries or device malfunctions is not known.^18^ It is not a research database and therefore limited to the contents of reports with variable quality, it is from a single country (the United States), and no event denominator is known. Moreover, granular clinical details and follow-up are unavailable, as such definitive device mortality causation cannot be determined. Each report is a snapshot of a device-related complication or malfunction. These limitations led to several events being classified as indeterminate as there was insufficient clinical data to classify the cause of death. In addition, these limit the evaluation for morbidity due to no long-term follow-up or limited outcomes. Therefore, this study focused on mortality events only and device malfunctions (eg, catheter breakage) or other non-mortality events were not examined, but future studies may benefit from examining mechanisms of failure.

## Conclusions

This study captures the highest number of mortality events related to AT in the literature and patterns of injury leading to death are identified. The most frequent and preventable causes of death were vascular perforation and right heart injury. Clot migration and aRHF were also important sources of mortality. While further large-scale randomized trials are ongoing, monitoring of the MAUDE database for this technology remains crucial for understanding mortalities during treatment.
